# Consensus Multilocus Sequence Typing Scheme for *Pneumocystis jirovecii*

**DOI:** 10.3390/jof6040259

**Published:** 2020-10-30

**Authors:** Lana Pasic, Lidia Goterris, Mercedes Guerrero-Murillo, Laszlo Irinyi, Alex Kan, Carolina A. Ponce, Sergio L. Vargas, M. Teresa Martin-Gomez, Wieland Meyer

**Affiliations:** 1 Molecular Mycology Research Laboratory, Centre for Infectious Diseases and Microbiology, Westmead Clinical School, Sydney Medical School, Faculty of Medicine and Health, The University of Sydney, Sydney 2006, Australia; lana.pasic@sydney.edu.au (L.P.); laszlo.irinyi@sydney.edu.au (L.I.); alex.kan@sydney.edu.au (A.K.); 2 Marie Bashir Institute for Infectious Diseases and Biosecurity, The University of Sydney, Sydney 2006, Australia; 3 Westmead Institute for Medical Research, Westmead 2145, Australia; 4 Microbiology Department, Hospital Universitari Vall d’Hebron (VHUH), 08035 Barcelona, Spain; lgoterris@vhebron.net (L.G.); mercedes.guerrero@vhir.org (M.G.-M.); mtmartin@vhebron.net (M.T.M.-G.); 5 Genetics and Microbiology Department, Universitat Autònoma de Barcelona (UAB), 08035 Barcelona, Spain; 6 Instituto de Ciencias Biomédicas (ICBM), Facultad de Medicina, Universidad de Chile, Santiago de Chile 8380453, Chile; cponce@med.uchile.cl (C.A.P.); svargas@med.uchile.cl (S.L.V.); 7 Westmead Hospital (Research and Education Network), Westmead 2145, Australia

**Keywords:** *P. jirovecii*, consensus MLST scheme, *β-TUB*, *CYB*, *mt26S* and *SOD*

## Abstract

*Pneumocystis jirovecii* is an opportunistic human pathogenic fungus causing severe pneumonia mainly in immunocompromised hosts. Multilocus sequence typing (MLST) remains the gold standard for genotyping of this unculturable fungus. However, the lack of a consensus scheme impedes a global comparison, large scale population studies and the development of a global MLST database. To overcome this problem this study compared all genetic regions (19 loci) currently used in 31 different published *Pneumocystis* MLST schemes. The most diverse/commonly used eight loci, *β-TUB*, *CYB*, *DHPS*, ITS1, ITS1/2, *mt26S* and *SOD*, were further assess for their ability to be successfully amplified and sequenced, and for their discriminatory power. The most successful loci were tested to identify genetically related and unrelated cases. A new consensus MLST scheme consisting of four genetically independent loci: *β-TUB*, *CYB*, *mt26S* and *SOD*, is herein proposed for standardised *P. jirovecii* typing, successfully amplifying low and high fungal burden specimens, showing adequate discriminatory power, and correctly identifying suspected related and unrelated isolates. The new consensus MLST scheme, if accepted, will for the first time provide a powerful tool to investigate outbreak settings and undertake global epidemiological studies shedding light on the spread of this important human fungal pathogen.

## 1. Introduction

*Pneumocystis jirovecii* is a major opportunistic pathogen, which can manifest into severe pneumonia, *Pneumocystis* pneumonia (PCP), in immunocompromised patients. PCP can cause interstitial lung disease, along with fever, coughs and dyspnea [1]. The incidence is still relatively high, especially in the developing world, for this underestimated fungus, with reported mortality rates ranging from 10% to 60% [2,3]. Besides causing isolated cases, *P. jirovecii* has been linked to nosocomial outbreaks affecting mainly solid organ transplant recipients with devastating consequences. Besides helping to establish epidemiological links among affected patients, allowing for paths of transmission to be mapped and index cases identified within hospital outbreaks, genotyping is an essential tool to achieve knowledge on more general aspects of the epidemiology of microorganisms.

Multilocus sequence typing (MLST) is currently the preferred standard for genotyping, due to the limited amounts of DNA being available from this unculturable fungus in clinical samples, its reproducibility, inexpensiveness and discriminatory ability [4]. However, unlike many other human pathogenic fungi, *Pneumocystis* has, as yet, no consensus typing scheme, which hinders the ability for global comparison of clinical isolates, since multiple various typing schemes are currently in use [5].

There are 19 coding and non-coding DNA regions which have been explored for *Pneumocystis* genotyping world-wide. The levels of allelic polymorphism fluctuate greatly between the used DNA regions, resulting in varying levels of discriminatory power among 31 schemes currently reported in the literature (see Table 1) [3,5,6,7,8,9,10,11,12,13,14,15,16,17,18,19,20,21,22,23,24,25,26,27,28,29,30,31,32,33,34,35,36,37,38,39,40,41,42,43,44,45,46,47,48,49,50,51,52,53,54,55,56,57,58,59,60,61,62,63,64,65,66,67,68,69,70,71]. The lack of standardisation limits the interpretation and comparison of different epidemiological datasets and studies. Molecular typing of *P. jirovecii* is further hampered by the fact that the fungus cannot be cultured in vivo, and hence the volume of DNA available for sequencing analysis is limited [72]. The DNA volume can be further depleted depending on the source and site of extraction, and if the patients are having a mild infection or if they are colonised carriers [73]. Therefore, the ability of a locus to be successfully amplified is equally as important as it is discriminatory power, when deliberating which loci to include in a standardised MLST scheme.

The aim of this study was to establish a consensus MLST scheme for *P. jirovecii,* taking into account the previously applied loci, to be used globally for *P. jirovecii* strain typing. Having a global consensus MLST scheme will allow for data exchangeability and comparisons of clinical isolates between laboratories, and the creation of an online world-wide MLST databank for *P. jirovecii* isolates.

## 2. Investigated Loci and Typing Schemes

To select the most appropriate genetic loci all published loci and respective *P. jirovecii* genotyping schemes have been evaluated. Since 1994 nineteen genetic loci, representing either single or multi-locus genes, have been used in diverse genotyping analyses of *P. jirovecii* (see Table 1). Due to the limited DNA amount extracted from *Pneumocystis* positive clinical samples, the loci were rated based on their previous published amplification and sequencing success rates, as well as the diversity revealed per locus and subsequent ability to discriminate between strains. 

### 2.1. Nuclear rRNA Gene Cluster

Firstly, the multi-copy nuclear rRNA gene cluster was studied. It consists of five components which have been amplified and sequenced previously, including the *18S* rDNA gene, the ITS1 region, the *5.8S* rDNA gene, the ITS2 region and the *26S* rDNA gene [6]. While the rDNA genes are highly conserved, the ITS regions show substantial diversity and as such have been used heavily for identification and genotyping of fungi [12]. The ITS1 and ITS2 regions have demonstrated the highest sequence variation among all loci of the rDNA gene cluster, as evident when sequenced as separate loci or in combination of the two regions (including the *5.8S* gene), using nested-PCR techniques [3]. This has resulted in over 120 unique genotypes for both ITS regions reported and submitted to GenBank [74]. The ITS1 and ITS2 regions were chosen over the other genes in the rDNA gene cluster due to their superior discriminative power.

### 2.2. Mitochondrial Genes

The mitochondrial large ribosomal subunit (*mt26S*) gene is involved in basic metabolic functions, with 15 copies within the genome [75]. This locus was selected as it has been considered to be a highly informative genetic marker due to its high variability between isolates, as well as being used as the main target world-wide for *P. jirovecii* detection and identification [76]. Another mitochondrial gene also selected was the *cytochrome b* gene, which contains approximately six copies per genome [75]. *Cytochrome b oxidase* gene (*CYB*) has been used widely within MLST genotyping of PCP infections and is increasingly commonly used within European hospitals and laboratories [41]. It has a reported high amplifying and sequencing success rate, although it offers a slightly lower variability than the *mt26S* locus. Although the mitochondrial small subunit (*mtSSU*) rDNA gene has over twice as many copies than *mt26S* gene, it has considerably reported lower variation over the *mt26S* gene, five unique genotypes compared to 25 [75,77]. Additionally, the locus has only appeared in six publications since 1998, when it was first sequenced for use in MLST. For all those reasons the *mtSSU* locus was not further considered for the consensus scheme.

### 2.3. Nuclear Genes

Finally, three nuclear genes, *β-tubulin* (*β-TUB*)*, dihydropteroate synthase* (*DHPS*) and *superoxide dismutase* (*SOD*) were also selected to be included in the study. *β-TUB*, is a single copy gene belonging to the *tubulin* coding gene family, which has been used for *Pneumocystis* identification and genotyping since the 1990′s and has been published within MLST schemes over 50 times [78]. Additionally, *β-TUB* has been used as a target locus for PCP diagnosis and is also part of the International Society of Human and Animal Mycology (ISHAM) MLST database (available online http://mlst.mycologylab.org), the only current MLST database specific to *Pneumocystis*, hence warranted further investigation [79]. *SOD* is a single copy gene encoding the production of the enzyme superoxide dismutase, which is commonly used in European studies as an efficient and discriminatory locus for genotyping [29,41]. *SOD* has a lower variation than *β-TUB*, but several studies have shown that these loci can be used to differentiate between colonised *Pneumocystis* (low burden levels) cases and high burden levels, such as in active PCP cases [80].

The final gene selected to be further investigated was *DHPS*, a highly studied locus due to nonsynonymous, point mutations within codons 55 and 57. These point mutations offer insights to trimethoprim-sulfamethoxazole (TMP-SMZ) resistance, due to the SMZ region of TMP-SMZ [81]. Drug resistances in *P. jirovecii* have been suggested by tracking the variations in *DHPS,* signifying an association between mutations and failure of prophylaxis with sulfa drugs [82]. Due to its high amplification success, along with the ability to give insight to resistance [70], *DHPS* is a key genetic region for many MLST studies. Despite its extensive use, low levels of genetic variation have been reported, with most studies reporting wild-type sequences being detected [83]. *DHPS* is also commonly genotyped together with *DHFR*, the *dihydrofolate reductase* encoding gene, as both enzymes are part of the folic acid pathway [46]. *DHFR* is inhibited by trimethoprim, nevertheless, information regarding the occurrence of mutations in *DHFR* are scarce, and also conflicting [70,82]. Studies have reported varying levels of *DHFR* mutations, from 2% to 60%, with no significant trends within global distributions, limiting its validity to be used for discrimination between samples [16,24,47,84]. Although this locus is still investigated within PCP treatment research, among the literature it shows slightly lower rates of variation than *DHPS*, and so would not add any further information when used alongside *DHPS* within a newly proposed MLST scheme.

The *major surface glycoprotein* (*msg*) gene, upstream conserved sequences (USC) genetic regions of the *kexin-like serine protease* gene (*Kex1*), *thymidylate synthase* gene (*TS*), *thioredoxin reductase* gene (*Trr1*), as well as the *5-enolpyruvylshikimate-3 phosphate synthase* activity (EPSP) region within the *arom* gene were not selected for further analysis within this study. These genetic regions displayed an inability to be adequately sequenced, as seen with the *msg* and *USC* genes [46,85]. The *Trr1*, *TS*, *Kex1* and *arom* gene loci showed a minimal sequence divergence due to being highly conserved housekeeping genes, and, as such, they are not suitable for MLST, as their discriminatory power is too low [13,41].

As a result of this theoretical analysis the following seven genetic loci have been selected for further practical exploration in this study: *β-tubulin* gene, *cytochrome b oxidase* gene, *dihydropteroate synthase* gene, internal transcribed spacer 1 (ITS1), internal transcribed spacer 2 (ITS2), *mitochondrial large ribosomal subunit rRNA* gene (*mt26S*, also known as *LSU-mt26S*) and *superoxide dismutase* gene.

## 3. Amplification Rate and Variation of Target Loci

Two cohorts of positive *Pneumocystis* DNA samples were independently subjected to amplification of the selected loci. The first cohort contained 44 bronchoalveolar lavage (BAL) samples which were PCP positive and were obtained from patients within Chilean hospitals between 2004 and 2014. The second cohort was composed of 23 oropharyngeal washes (OW) and 63 BAL samples from a single centre in Spain, between 2014 and 2018, with 35 positive PCP cases and 51 colonisations.

*P. jirovecii* PCP was diagnosed either by Grocott-Gomori’s methenamine silver stain or direct immunofluorescence (Meridian Bioscience, Inc.) [86]. Samples were processed inside a biosafety cabinet using sterile precautions to avoid contamination. They were homogenised with a sterile pipette and a 200 μL aliquot was used for DNA extraction with the QIAamp^®^ DNA Blood Mini kit (Qiagen). *P. jirovecii* was confirmed via PCR of the mitochondrial large subunit rRNA using the primers pAZ102-E and pAZ102-H [87]. Negative controls were included to monitor for cross-contamination during DNA extraction and purification. An internal control using the *human β-globin* gene [79] was used in each sample to detect false negatives. Each sample was run undiluted and as a 1/5 dilution. 

The samples were subjected to amplification to assess the effectiveness of the loci in a practical setting, within a range of *Pneumocystis* samples. To yield higher success rates the PCR primers and the associated amplification protocols were optimised from previously published conditions. Genes were amplified in volumes of 25 μL per PCR reaction, using 10X buffer (100 mM Tris-HCL, pH 8.3, 500 mM KCl, 15 mM MgCl_2_ and 0.01% *w*/*v* gelatine), 50 nM MgCl_2_, 2 mM dNTPs, 10 ng/μL of each primer, 5 U/µL BIOTAQ DNA (Bioline) polymerase and 10 µL of genomic DNA. The optimised primers and amplification conditions suggested to be used for the new consensus MLST scheme are shown in Table 2. The amplification conditions which have been used to amplify the loci not included in the new consensus MLST scheme are shown in the Supplementary Table S1.

Bidirectional sequencing was performed at Macrogen Inc., Seoul, South Korea. The obtained sequences where then assembled and edited using the software package Sequencher ver. 5.4.6 (Gene Codes Corporation). The cleaned-up sequences for each locus were aligned with the program CLUSTALW [88] part of the software MEGA version 10.1 [89] and compared to reference sequences listed in Table 2 and Supplementary Table S1. Allele types were named with respect to previously the published nomenclature [5,41].

**Figure 1:**
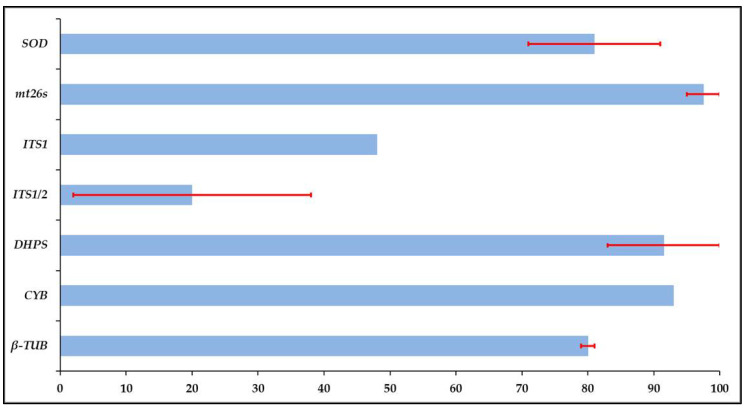
Average amplification and sequencing rates for all targeted genetic loci. Calculations are based on the combined mean amplification and sequencing rates of both cohorts and are expressed as percentages. Error bars indicate the standard deviation.

The obtained amplification and sequencing success rates varied widely, with the nested PCR of the ITS1/2 locus being the lowest, with 2% for the Chilean isolates and only 38% of the Spanish isolates. *DHPS* and *mt26S* loci had the highest, which amplified 83% for the Spanish and 100% for the Chilean isolates and 95% of the Spanish and 100% of the Chilean isolates, respectively. Simple PCR of the ITS1 region with the newly designed primers amplified 47% of the Chilean isolates, although the Spanish were not amplified with this primer. This was then followed by the *β-TUB* locus, which amplified 80% of the Spanish and 78% of the Chilean isolates, the *SOD* locus, which amplified 71% of the Spanish and 91% of the Chilean isolates, and the *CYB* locus, which amplified 94% and 93% of both Spanish and Chilean isolates, respectively. Average amplification rates of the two cohorts are seen in Figure 1. The Fisher exact test statistic value indicated no significant differences between the individual cohort amplification rates for *β-TUB*, *CYB* and *mt26S*, and a significant result at *p* < 0.05 using the Fisher’s exact test [90] for the *DHPS*, *SOD* and ITS1/2 loci (for all raw data see Supplementary Table S2).

**Figure 2:**
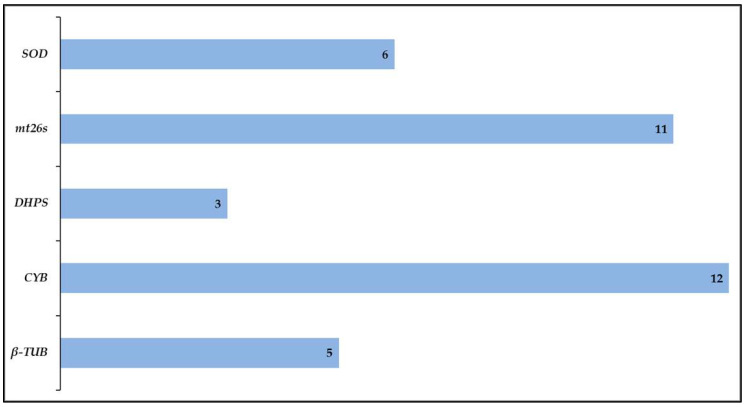
Number of allele types identified amongst all studied isolates.

The genetic loci were then assessed for their ability to discriminate between different strains, as a high variability within individual loci will directly increase the discriminatory value of the consensus scheme. Based on the PCR performance the ITS1/2 and ITS1 loci were not further analysed due to their poor amplification rates. The mitochondrial genes, *mt26S* and *CYB*, were found to have the highest variation, followed by *β-TUB* and *SOD,* as judged from the number of unique allele types obtained. The *DHPS* locus showed the least amount of variation, with the vast majority of alleles corresponding to the wild type and only two variants having been found across the entire collection of samples (Figure 2). A new database of all allele and sequence types has been established at http://mlst.mycologylab.org for the newly proposed consensus *P. jirovecii* MLST scheme.

Based on the above results, showing a superior amplification rate and sequence quality, and high discriminatory power, the following four loci: *β-TUB*, *CYB*, *mt26S* and *SOD* were chosen for inclusion in the proposed new consensus *P. jirovecii* MLST scheme.

## 4. Case Study: Assessing the Ability to Discriminate between Clinical Isolates

**Figure 3:**
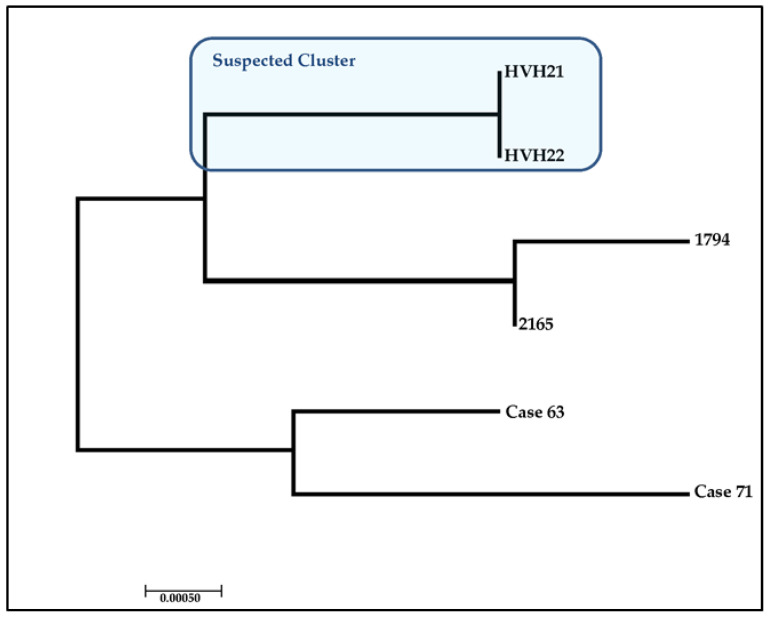
Phylogenetic tree of six *P. jirovecii* isolates (patients from Table 3) used in the case study to show the discriminatory power of the new consensus *P. jirovecii* MLST scheme, obtained by maximum likelihood analysis with the general time reversible (GTR) model with RaxML (version 7.2.8) using RaxmlGUI 1.1 [91], part of the software package MEGA ver. 7.0 [92].

To access the efficiency to identify related and to differentiate between unrelated *P. jirovecii* isolates these four loci were then used to genotype six positive *Pneumocystis* samples representing a potential cluster (two epidemiologically linked isolates) and four independent cases. Allelic profiles were assigned to each sample using the newly developed MLST database available online at http://mlst.mycologylab.org (Table 3) confirming the suspected to be related isolates and successfully separated all four suspected unrelated isolates (Figure 3). The two suspected related isolates showed identical MLST profiles corresponding both to the ST21. The four suspected unrelated isolates had all unique MLST profiles, ST2, ST7, ST42 and ST44, and where also different from the two related isolates (Table 1 and Figure 3). Cross contamination between samples was ruled out as samples were analysed on different days and results were checked by resequencing a second aliquot.

## 5. Discussion

Genotyping of *P. jirovecii* is vital for the advancement of understanding of the biology, pathogenesis, epidemiology, prophylaxis and treatment regimen of this human pathogen, but more specifically it is vital to help manage, contain and prevent nosocomial clusters. With a rise of nosocomial outbreaks since the early 2000s, hospitals have recorded catastrophic consequences of PCP outbreaks, with up to 83% of reported outbreaks being described within organ transplant wards, as well as in patients with haematological malignancies and connective tissue diseases [93]. Large graft failure and over 50% casualties in wards has been reported from single outbreaks, demonstrating the severity of this underestimated disease [94].

An effective way to investigate epidemiological links is through the creation of transmission maps by combining molecular typing along with studying patient encounters and interactions within the hospital [5]. However, the fact that there are currently 19 genetic regions being used in 31 different typing schemes for *P. jirovecii* limits effective epidemiological studies. The lack of a consensus scheme directly inhibits the ability to compare results, polymorphic strains, and epidemiological data between research centres, and hinders the possibility of establishing global databases and conducting large-scale population studies.

To overcome these limitations, a comprehensive study of all genes and schemes was herein undertaken to assess which loci and which combination of loci would allow for the development of the most practical, efficient and discriminatory scheme. Previous studies looked at the performance of various schemes to suggest a possible consensus scheme, but none have since been formally brought forward as a suggested universal scheme [3]. Maitte et al. 2013 [5], in their review suggested that an eight loci scheme provided the most powerful genotyping results, but this is only possible theoretically due to the limited amount of DNA available from *P. jirovecii* in clinical samples. There are no reliable methods to culture the *P. jirovecii* in vitro, therefore the DNA amount which comes directly from clinical samples is limited [7]. The volume is then further limited depending on the fungal load as well as the source of the specimen [95]. HIV positive patients have high fungal loads, whilst colonised carriers and HIV-negative PCP patients carry lower levels [96]. Bronchoalveolar lavage specimens are the preferred sample, yielding the highest sensitivity due to a greater fungal concentration and also yielding an acceptable negative predictive value [97]. The same studies have shown that the less invasive method, induced sputum (IS), showed comparable levels of fungal burden as BAL, followed by oropharyngeal washes, and then to a lesser extent nasopharyngeal aspirates, and nasal swabs [93,95]. It is therefore vital to have a typing system, consisting of as few genetic loci as possible, but being able to detect and amplify specimens with low levels of fungal burden, as this is often the first limiting step when undergoing *Pneumocystis* genotyping analysis.

On the other side, having too few loci also poses a problem, since lowering the number of loci in a scheme then decreases the discriminatory power and the ability to distinguish between closely related strains. Studies have shown that often schemes with less than three loci do not have enough variation to accurately genotype, as such the Hunter (H)-index has been used in multiple studies to demonstrate the discriminatory power of a scheme [5]. The H-index should not be the only determining factor of an effective scheme as it is highly variable depending on the number of isolates being tested, but it has been a useful tool to help predict the estimated discriminatory power of a scheme [98]. A H-index of 0.95 or higher is considered a suitable cut-off benchmark for MLST schemes, and the review by Maitte et al. 2013 [5] showed that there were no genetic regions which could work individually or paired with another, that would meet this cut-off [19]. As seen in Table 1, there are multiple schemes used in genotyping which are comprised of only two loci, casting a shadow of doubt on their results and generated epidemiological data of these studies, further reinforcing the need for standardisation amongst the *Pneumocystis* scientific community.

As a result, eight genetic regions were explored in this study as potential loci which could be included in a global consensus scheme, by analysing their amplification ability and demonstrating their discriminatory power. The whole ITS region is widely reported to have the most variability within *P. jirovecii* and is highly useful when identifying and genotyping other invasive fungi [74]. Unfortunately, it also has been reported to have a high amplification failure when applied to *P. jirovecii* [59], which was also evident in the current study, as ITS1 alone and ITS1/2 regions showed to have the lowest amplification capability, unable to amplify and successfully sequence more than 50% of the studied isolates for either primer set. Studies have reported that using nested-PCR instead of conventional, single-round PCR can help to overcome the lack of amplification for the ITS region [3,99]. This was not the case in the herein reported study, as the nested-PCR protocol for the ITS1/2 regions performed considerably worse than single round PCR for the ITS1 region within the Chilean cohort. Due to the lack in amplification success, both in this study and others, no loci from the nuclear rRNA gene cluster were further considered for involvement in a new consensus MLST scheme.

Conversely to the ITS region, *DHPS* amplification was highly successful achieving a global 91.5% amplification success, with minor variations that can be attributed to differences associated to the spectrum of clinical settings covered, ranging from colonisations to infections. *DHPS* is heavily used among *P. jirovecii* genotyping and has been consistently used since the late 1990s in diverse schemes. Despite this, within the reported literature, *DHPS* does not reveal much variation, as such most often wild-types are reported, carrying no informative data to discriminate between strains, showing a H-index of 0 in previous studies [5,100,101]. *DHPS* has demonstrated effectiveness when exploring TMP-SMZ resistance, by showing mutations at the 55 and 57 codons [102]. Population studies using the *DHPS* locus have effectively been able to track additional sulfa pressure in geographical regions with or without widespread use, and as such accurately predict or identify sulfamethoxazole resistance within these regions [103,104]. In this study, only two allele types have been identified, with most of the isolates showing the wildtype, showing regional differences in the distribution of wildtype and allelic variants, subject to further studies. The mutations can be used to give an insight in the widespread use of TMP-SMZ, and for a further comparison between other geographical locations heavily using SMZ but not showing resistance within the community. From a genotyping perspective, the *DHPS* locus, however, does not offer enough variation to be considered as a useful locus within a consensus scheme, and was therefore no longer considered within this study. Despite its drawback to sufficiently discriminate between isolates, researchers should maintain *DHPS* typing for assessing prophylaxis and treatment resistance within populations, and *DHPS* can further be explored for its use as an identifying genetic target in PCP diagnosis due to its successful amplification properties.

Two schemes which use the loci explored in this study and are predominant in the literature are: The official scheme promoted by ISHAM [3], consisting of *β-TUB*, *DHPS*, ITS1 or ITS1/2 and *mt26S*; and a French scheme first proposed by Maitte et al. 2013 [5], consisting of *CYB*, *mt26S* and *SOD*. The ISHAM scheme is highly discriminatory, when applied to both herein studied cohorts it resulted in an H index of >0.98. for both cohorts. The H index was calculated as per Hunder et al. 1988 [98]. Due to different ITS regions explored, ITS1 in the Chilean cohort was able to identify 23 unique sequence types using this scheme, and ITS1/2 in the Spanish cohort attained 14 sequence types. The Maitte et al. 2013 [5] scheme has been reported to have a H-index above 0.95, but this index fell below the threshold when larger sample sizes were considered. H indexes ≤ 0.945 were attained when herein applied to the Chilean and Spanish cohorts, with 33 unique ST detected.

Since the *DHPS* and ITS are not appropriate loci to be used in MLST schemes, the ISHAM scheme could not be promoted for universal application, and neither could the Maitte et al. 2013 [5] scheme, due to inconsistencies with sub-optimal levels of discrimination. *β-TUB*, *CYB*, *mt26S* and *SOD* genetic regions were all individually effective in amplification, but unable to individually discriminate effectively enough, therefore a new consensus MLST scheme comprising of these four loci is herein proposed.

When applied to both cohorts, the new MLST scheme was able to discover a total of 38 unique sequence types, with a combined H-index of 0.975, which is well above the discriminatory cut-off margin for any useful MLST typing scheme.

As this new MLST scheme had not been previously used, the next step was to explore the effectiveness of the scheme to successfully individual *P. jirovecii* isolates. The new MLST scheme was applied to two suspected epidemiologically linked isolates and four isolates for which clinical metadata suggested no relationship, to determine whether it would be discriminatory enough to distinguish the isolates appropriately. The MLST analysis revealed a distinct cluster, showing the genotype ST1, consisting of two patients, named HVH21 and HVH22. The identical sequences of patient HVH21 and HVH22 confirmed the suspected fact of relatedness, which is based on the metadata of the patients, which suggested a possible nosocomial cross-transmission. The cluster involved an HIV positive colonised patient and a colonised lung transplant recipient, both with a positive BAL, obtained three days apart. Transmission could have taken place at the radiology department, which both had attended on the same day. The other four epidemiologically unlinked isolates were successfully separated from the cluster and revealed each a unique sequence type, ST7, ST13, SR18 and ST21, which was expected as they were obtained from patients from different geographic regions, including Australia, New Zealand and Chile. With the Chilean isolates originating from patients from different Chilean health centres (public and private) three years apart. As such, the newly established consensus MLST scheme has demonstrated its ability to discriminate appropriately between *P. jirovecii* isolates, making it a powerful tool to identify identical strains in settings with many associated cases, such as in an outbreak situation. However, the obtained typing data should always be complemented with a clinical history as good as possible to trace back the origin.

The herein obtained results demonstrated that the new MLST scheme consisting of *β-TUB*, *CYB*, *mt26S* and *SOD* (Table 2) has a much higher amplification rate and an efficient discriminatory power to be applied for genotyping of *P. jirovecii* isolates from clinical samples with high and low fungal burdens, including disease causing and colonising isolates.

Promoting this novel MLST scheme as a global consensus scheme will for the first time standardise MLST for the human pathogen *P. jirovecii* and set up the basis of a substantial improvement in understanding the relationship between clinical *P. jirovecii* isolates. This will allow real-time genotyping of current infected patients and suspected colonised carriers to be now undertaken to improve the understanding of transmissions and the effect colonised carriers have on nosocomial spreads. Further, this will influence public health approaches for preventing nosocomial infections, in especially high-risk patients, such as those recovering from organ transplantation in close approximate in hospital wards.

Since there is no current database, outside of the ISHAM MLST database [3], available online at http://mlst.mycologylab.org/, it is difficult to find all currently published allele types and the lack of standardisation across global centres often cause confusion, even when attempting to compare allele types of a certain locus. The new global database, also placed at http://mlst.mycologylab.org, established herein will improve the nomenclature of allele types and sequence types and makes it easier for researchers and clinician to have one source of information for all genotyping data. The herein standardised MLST scheme will enable the establishment of such a global database, which can be used by all clinical diagnostic and research centres to deposit metadata and sequences, allowing to compare global specimens, something which was not previously possible.

## 6. Conclusions

In conclusion, this study demonstrates the importance of a consensus MLST scheme for *P. jirovecii* genotyping and the formation of a global database in expanding the understanding of this important human pathogenic fungus. Based on the previous schemes and the evidence in this study, a novel MLST for the genotyping of *P. jirovecii*, consisting of four genetic regions: *β-TUB*, *CYB*, *mt26S* and *SOD* is proposed. This combination of loci maximises the likelihood for amplification and adequate discrimination of isolates over previously used schemes and will aid hospitals in drawing conclusions about interhuman transmission between patients, and hopefully minimise or early detect nosocomial outbreaks.

## Figures and Tables

**Table 1:** Published genetic loci used in *P. jirovecii* genotyping, corresponding multilocus sequence typing (MLST) schemes and obtained allele and sequence types. MLST schemes described are listed chronologically, followed by the respective publications using the specific scheme. The third column indicates the total number of isolates included in the study, followed by the fourth column, which indicates the number of isolates which were able to be successfully sequenced by the study. The fifth column lists the total number of sequence types identified, with the following columns listing the number of allele types found for each genetic locus.

Schemes (Included Loci) and Reference	Country	Total # of Samples	# of Samples Sequenced	# of Sequence Types	Genetic Locus																			
					*5.8S*	*18S*	*23S*	*26S*	ITS1	ITS2	ITS1/2	*msg*	*mt26S*	*β-TUB*	*TS*	*arom/EPSP*	*mtSSU*	*DHPS*	*UCS*	*Kex1*	*CYB*	*SOD*	*DHRF*	*TRR1*
**Scheme 1 (*5.8S, 18S, 26S,* ITS1, ITS2)**																								
[6]	USA	15	15	6	1	1		1	**2**	**3**	**NG**													
[7]	GBR	24	24	NG	1	1		1	**5**	**7**	**NG**													
**Scheme 2 (ITS1, ITS2, *msg, mt26S*)**																								
[8]	USA	15	15	NG					**NG**	**NG**	**NG**		**NG**											
[38]	IND	180	29	NG					**NG**	**NG**	**NG**		**NG**											
**Scheme 3 *(26S, β-TUB,* ITS1, *mt26S*)**																								
[9]	CHE	11	11	NG				3	**3**				**3**	**2**										
[20]	EUR	212	212	6				6	**3**				**4**	**3**										
[19]	EUR	91	91	28				NG	**NG**				**NG**	**NG**										
[33]	DEU	7	7	2				NG	**NG**				**NG**	**NG**										
[42]	DEU	20	14	NG				2	**4**				**4**	**1**										
[43]	CHE	19	7	1(+)				2	**4**				**3**	**2**										
[50]	DE	18	18	NG				2	**3**				**3**	**2**										
[53]	GBR	670	31	NG				NA	**5**				**4**	**NA**										
[54]	FRA	13	10	3				2	**2**				**3**	**1**										
**Scheme 4 (ITS1, ITS2, *mt26S*)**																								
[10]	USA	15	15	6					**4**	**3**	**7**		**4**											
[11]	SWE, FRA	7	7	NG					**4**	**4**	**NG**		**3**											
[48]	GBR	27	27	NG					**NG**	**NG**	**2**		**3**											
**Scheme 5 (*5.8S,* ITS1, ITS2, *mt26S, TS*)**																								
[12]	FRA, ITA	20	18	NG	6				**3**	**3**	**10**		4		1									
**Scheme 6 (*arom,* ITS1, ITS2, *mt26S, mtSSU*)**																								
[13]	NLD	6	6	NG					**NG**	**NG**	**9**		3			1	2							
**Scheme 7 (ITS1, ITS2)**																								
[14]	GLO	207	207	NG					**15**	**14**	**NG**													
[18]	JPN	24	24	NG					**11**	**11**	**NG**													
[31]	ZAF	20	20	NG					**11**	**13**	**NG**													
[49]	SWE	64	64	12					**10**	**12**	**NG**													
**Scheme 8 (*DHPS*, ITS1, ITS2, *mt26S*)**																								
[15]	FRA	14	14	NG					**NG**	**NG**	**NG**		**NG**					**NG**						
[39]	PRT, ESP	108	✕	NG					**12**	**10**	**NG**		**4**					**4**						
[44]	AUS	68	68	NG					**8**	**9**	**16**		**2**					**2**						
**Scheme 9 (*DHFR, DHPS*)**																								
[16]	USA	37	37	NG														**4**					2	
[32]	FRA	33	33	NG														**3**					2	
[40]	THA	29	18	NG														**3**					3	
[71]	USA	13	13	NG														**2**					2	
[70]	COL	98	45	NG														**4**					2	
**Scheme 10 (*DHPS, mt26S*)**																								
[17]	USA	324	191	14									**4**					**4**						
[26]	ESP	255	79	NG									**4**					**4**						
[34]	ESP	50	12	NG									**4**					**1**						
[35]	USA	442	❋	NG									**4**					**4**						
[45]	ESP	60	19	NG									**3**					**1**						
[51]	ITA	67	⚑	NG									**4**					**3**						
**Scheme 11 (*26S, β-TUB, DHPS,* ITS1, *mt26S*)**																								
[21]	USA	22	22	10				2	**5**				**4**	**2**				**3**						
**Scheme 12 (*26S, β-TUB,* ITS1, ITS2, *mt26S*)**																								
[22]	ITA	25	18	15				4	**6**	**6**			**4**	**3**										
**Scheme 13 (*DHPS,* ITS1, ITS2)**																								
[23]	USA	57	37	NG					**6**	**7**	**NG**							**3**						
[27]	PRT	43	43	NG					**15**	**14**	**17**							**2**						
[36]	ITA	261	174	NG					**NG**	**NG**	**9**							**3**						
**Scheme 14 (*CYB, DHFR, DHPS*)**																								
[24]	JPN	34	34	NG														**4**			**2**		1	
**Scheme 15 (*DHPS,* ITS1, ITS2, *mtSSU*)**																								
[25]	GBR	2	2	NG					**NG**	**NG**	**2**						1	**1**						
**Scheme 16 (*DHPS, mt26S, mtSSU*)**																								
[28]	GBR, ZWE	51	30	NG									**3**				4	**2**						
**Scheme 17 (*DHPS, mtSSU, mt26S, SOD*)**																								
[29]	GBR	16	16	NG									NG				NG	**NG**				**NG**		
[30]	NG	76	76	15									**4**				4	**4**				**4**		
**Scheme 18 (*DHFR, DHPS,* ITS1, ITS2)**																								
[37]	PRT	68	68	NG					**NG**	**NG**	**19**							**4**					4	
**Scheme 19 (*UCS, Kex1*)**																								
[39]	PRT	87	35	NG															4	4				
**Scheme 20 *(CYB, DHFR, DHPS, mt26S, SOD)***																								
[46]	PRT	102	78	NG									**9**					**6**			**3**	**6**	6	
**Scheme 21 (*β-TUB, CYB, DHFR, DHPS, mt26S, TRR1, TS, SOD*)**																								
[47]	PRT	70	►	48									**5**	**3**	⌘			**3**			**7**	**4**	3	⌘
**Scheme 22 *(β-TUB, DHPS,* ITS1/2, *mt26S*)**																								
[3]	AUS	11	11	2							**4**		**2**	**2**				**1**						
[55]	AUS	48	48	4							**2**		**2**	**1**				**1**						
[57]	AUS	7	7	NG							**NG**		**NG**	**NG**				**NG**						
**Scheme 23 (*mt26S, mtSSU*)**																								
[52]	FRA, CUB, ESP	75	75	NG									**5**				3							
**Scheme 24 (26S, *β-TUB, CYB, DHFR, DHPS,* ITS1, *mt26S, SOD*)**																								
[5]	FRA	23	23	NG				7	**9**				**4**	**2**				**1**			**7**	**3**	3	
**Scheme 25 *(26S,* ITS1, ITS2, *mt26S*)**																								
[56]	DNK	22	18	3				NG	**NG**	**NG**	**NG**		**NG**											
**Scheme 26 (*CYB,* ITS1, *mt26S, SOD*)**																								
[58]	FRA	37	32	NG					**NG**				**NG**								**NG**	**NG**		
**Scheme 27 (*β-TUB, CYB, DHFR, mt26S, SOD*)**																								
[59]	BEL	20	20 ^	NG					**NA**				**4**	**2**							**4**	**2**	2	
**Scheme 28 (*CYB, mt26S, SOD*)**																								
[60]	FRA	24	◎	14									**6**								**5**	**3**		
[61]	FRA	32	32	18									**22**								**14**	**4**		
[62]	FRA	7	7	NG									**4**								**3**	**2**		
[63]	POL	17	◉	8									**13**								**6**	**2**		
[66]	FRA	192	35	17									**11**								**5**	**2**		
[67]	TUR	31	26	6									**4**								**5**	**3**		
[68]	REU, GUF, FRA	47	47	23									**5**								**9**	**3**		
**Scheme 29 (*23S, 26S, DHPS*)**																								
[64]	BRA	30	30	5			3	2										**1**						
**Scheme 30 (*DHFR, DHPS, mt26S*)**																								
[65]	IND	37	37	13									**3**					**3**					2	
**Scheme** **31 (*CYB, DHPS, mt26S, SOD*)**																								
[69]	POL	72	N/A	N/A									**3**					**1**			**3**	**2**		

Loci in black did not match diversity criteria for further consideration. Loci in blue indicate loci investigated in this study, but not included in the newly proposed MLST scheme. Loci in green indicate loci suggested from this study for the newly proposed consensus MLST scheme. NG = Information not given; NA = No amplification recorded; and 1(+) = Study only listed the sequence types (STs) for test isolates, 5 ST were identified when the controls are included. ✕ = 91 samples amplified for *dihydropteroate synthase* (*DHPS*) and 68 for Internal Transcribed Spacer (ITS); ❋ = 100% for *mitochondrial large ribosomal subunit* (*mt26S*) and 53% for *DHPS*; ⚑= 67 for *mt26S* and 21 for *DHPS*; ► = *mt26S* 100%, *cytochrome b oxidase* gene (*CYB*) 61%, *superoxide dismutase* (*SOD*) 74%, *β-tubulin (β-TUB*) 49%, *dihydrofolate reductase* gene (*DHFR*) 91%, *DHPS* 96%, *thioredoxin reductase* gene (*Trr1*) and *thymidylate synthase* gene (*TS*) 36%; ⌘ = Null sequence divergence, not included in study further for genotyping; ^ = ITS no amplification; ◎ = 78% *SOD*, 96.4% *mt26S* and 82.1% *CYB*; ◉ = 17/17 *mt26S* and *CYB*, 5/17 *SOD.* Country codes are according to ISO 3166-1 alpha-3.

**Table 2:** Primer information for the loci of the novel MLST scheme.

Locus	Primer Name	Ref.	Nucleotide Sequence	Product Size (Base Pairs)	PCR Conditions
*β-TUB*	PneumoβTub_F	-	5′-TCATTAGGTGGTGGAACGGG-3′	303	95 °C 3 min; 45 cycles: 94 °C 30 s, 60 °C 45 s, 72 °C 45 s; 72 °C 7 min
	PneumoβTub_R		5′-ATCACCATATCCTGGATCCG-3′		
*SOD*	MnSODFw	5	5′-GGGTTTAATTAGTCTTTTTAGGCAC-3′	602	
	MnSODRw		5′-CATGTTCCCACGCATCCTAT-3′		
*CYB*	CytbFw	5	5′-CCCAGAATTCTCGTTTGGTCTATT-3′	579	95 °C 3 min; 45 cycles: 94 °C 30 s, 55 °C 45 s, 72 °C 45 s; 72 °C 7 min
	CytbRw		5′-AAGAGGTCTAAAAGCAGAACCTCAA-3′		
*mt26S*	PneumoLSU_F	-	5′-TCAGGTCGAACTGGTGTACG-3′	297	
	PneumoLSU_R		5′-TGTTCCAAGCCCACTTCTT-3′		

**Table 3:** Allele types and sequence types of two related and four unrelated *P. jirovecii* isolates. Colours indicate the different allele types per genetic locus.

Strain Number	Country of Origin	Date of Sample	*β-TUB*	*CYB*	*mt26S*	*SOD*	Sequence Type
HVH21	Spain	Jan 2015	2	1	3	1	ST21
HVH22	Spain	Jan 2015	2	1	3	1	ST21
Case 63	Australia	Dec 2016	1	3	1	4	ST42
Case 71	New Zealand	May 2017	4	2	2	4	ST44
1794	Chile	Feb 2011	2	5	4	1	ST7
2165	Chile	Oct 2014	2	2	4	5	ST2

## Supplementary Materials

The following are available online at https://www.mdpi.com/2309-608X/6/4/259/s1, Table S1: Primer information for the initially tested but finally not selected loci. Table S2: Amplification rates of all tested loci for both cohorts.
