# Supplementary material for: Consensus Multilocus Sequence Typing Scheme for Pneumocystis jirovecii
Source: J Fungi (Basel). 2020 Oct 30;6(4):259. doi: 10.3390/jof6040259 (PMC7711988; doi:10.3390/jof6040259)
Supplement: Supplementary file 1 [file jof-06-00259-s001.zip › Table S2.docx]

Supplementary **Table S2:** Amplification rates of all tested loci for both cohorts.

| **Locus** | **Chilean Amplification Rates** | | **Spanish Amplification Rates** | | **Average Amplification** | **Fisher Exact Test Statistical Value, p < 0.05** | |
| --- | --- | --- | --- | --- | --- | --- | --- |
|  | **Raw Data** | **Percentage** | **Raw Data** | **Percentage** | **Percentage** | **Not Significant** | **Significant** |
| *β-TUB* | 41/52 | 79 | 69/86 | 80 | 80 | 0.831 |  |
| *CYB* | 41/44 | 93 | 80/86 | 93 | 93 | 1 |  |
| *DHPS* | 44/44 | 100 | 71/86 | 83 | 91.5 |  | 0.00024 |
| ITS1/2 | 1/44 | 2 | 18/48 | 38 | 20 |  | 0 |
| ITS1 | 21/44 | 48 | - | - | 48 |  |  |
| *mt26s* | 44/44 | 100 | 82/86 | 95 | 97.5 | 0.2992 |  |
| *SOD* | 40/44 | 91 | 61/86 | 71 | 81 |  | 0.0133 |
