# Supplementary material for: Consensus Multilocus Sequence Typing Scheme for Pneumocystis jirovecii
Source: J Fungi (Basel). 2020 Oct 30;6(4):259. doi: 10.3390/jof6040259 (PMC7711988; doi:10.3390/jof6040259)
Supplement: Supplementary file 1 [file jof-06-00259-s001.zip › Table S1.docx]

Supplementary **Table S1:** Primer information for the initially tested but finally not selected loci.

| **Locus** | **Primer Name** | **Reference** | **Nucleotide sequence** | **Product size (base pairs)** | **PCR Conditions** |
| --- | --- | --- | --- | --- | --- |
| ***DHPS*** | PnuemoDHPS-F | - | 5´-GCGCCTACACATATTATGGCCATTTTAAATC-3´ | 705 | 95°C 3 min; 45 cycles: 94°C 30s, 55°C 45s, 72°C 45s; 72°C 7min |
|  | PnuemoDHPS-R |  | 5´-ACCTTCCCCCACTTATATC-3´ |  |  |
| **ITS1** | PneumoITS-F | - | 5’-CCATTGCTGGAAAGTTGATCA-3’ | 722 |  |
|  | PneumoITS-R |  | 5’-TCGCCGTTACTAAGGGAATC-3’ |  |  |
| **ITS1/2**  **1^st^ round** | 1724F2 | 3 | 5´-AGTTGATCAAATTTGGTCATTTAGAG-3 | N/A | 96°C 5 min; 25 cycles: 94°C 1 min, 60°C 1 min, 72°C 45s; 72°C 7 min |
|  | ITS2R |  | 5´-CTCGGACGAGGATCCTCGCC-3´ |  |  |
| **2^nd^ round** | ITS1F2 |  | 5´-CGTAGGTGAACCTGCGGAAGGATC-3´ | 549 | 96°C 5 min; 20 cycles: 94°C 1 min, 56°C 1 min, 72°C 45s; 72°C 7 min |
|  | ITS2R1 |  | 5´-GTTCAGCGGGTGATCCTGCCTG-3´ |  |  |
